# A study on the influence of personality characteristics on household charitable donation behavior in China

**DOI:** 10.1371/journal.pone.0284798

**Published:** 2023-05-17

**Authors:** Kai Zhang, Bin Cao, Ya Zhang, Yawen Han

**Affiliations:** 1 WeBank Institute of Fintech, ShenZhen University, ShenZhen, Guangdong Province, China; 2 China Merchants Bank, ShenZhen, Guangdong Province, China; 3 Huazhong University of Science and Technology, Wuhan, Hubei Province, China; Imperial College London, UNITED KINGDOM

## Abstract

Using the data of the 2018 China Family Panel Studies (CFPS), this paper empirically tests the impact of the "Big Five" personality characteristics on household charitable donation behavior. The benchmark regression results show that after controlling the individual characteristics and family characteristics of the household heads, the conscientiousness and openness of the household heads have a significant positive impact on the social donation behavior of the family. On this basis, this paper takes the openness personality as an example, selects the identification strategy of processing effect, and tests the robustness of the causal effect of personality on household donation behavior. The openness personality has a significant positive impact on household external donation behavior. In the further study, it is found that with the improvement of the level of household charitable donation, the positive effect of the household head ’s openness personality on household charitable donation behavior is gradually weakening; The influence of openness personality on household charitable donation has the nonlinear characteristics of "marginal effect" increasing and obvious life cycle characteristics.

## Introduction

Under the concept of common prosperity, the current distribution system framework followed by China is "not only to achieve stable and orderly development of common prosperity, but also to take into account the relationship between efficiency and fairness, and finally to achieve the reasonable construction of the basic system of economic distribution coordination (distribution-redistribution-three distribution)". Since the third distribution is completed by residents or entrepreneurs on a voluntary basis [1], encouraging the development of social public welfare undertakings such as charitable donations is, to a certain extent, regarded as a necessary measure to promote the current economic development into a new stage. At present, it is found that the willingness and level of donation of Chinese households are not high, the amount of donation is low, and the donation is temporary [2, 3]. How to further promote the charitable donation behavior of residents’ families, improve the level of donation, reasonably realize the redistribution of wealth, and realize the common prosperity of the whole people, is a question worthy of consideration.

The family can be regarded as a fundamental unit of prosocial donation behavior. The Chinese-style family has the typical oriental characteristics of intergenerational inheritance. Exploring the charitable donation activities of Chinese families will help to further activate the potential of charitable donation of Chinese residents and open a new situation for the development of philanthropy and the third distribution. In existing studies, household donation behavior is usually affected by many factors such as individual characteristics [4] and family characteristics [5]. In addition to these two characteristics, whether personality characteristics will also affect household donation behavior is a question worthy of discussion. Based on the above background, this paper explores residents’ charitable donation behavior from both theoretical and empirical perspectives.

In the existing research, the "Big Five" personality classification method is widely accepted and widely used at present, which generally divides personality characteristics into five types: openness, conscientiousness, extraversion, agreeableness and neuroticism [6]. Most of them expound the relationship between personality characteristics and economic indicators from the perspective of economics, sociology and psychology [7, 8], but few literatures have studied the relationship between personality characteristics and donor behavior, especially in the context of China, there is no empirical analysis of the “Big Five” personality characteristics on donor behavior.

Therefore, based on the existing research results of personality economics, this paper uses the special questionnaire data of the "Big Five" personality characteristics in the 2018 CFPS. This paper studies the relationship between the "Big Five" personality characteristics and household charitable donations from an empirical perspective. This paper focuses on the openness personality characteristics, and uses the maximum likelihood estimation method to respond to the effect model established in this process, so as to test the impact of personality characteristics on Chinese household charitable donation behavior. Specifically, the study analyzed the impact of personality characteristics on different types of external donations, used quantile regression to study the impact of personality characteristics on different levels of household charitable donations, and used threshold regression to study the non-linear characteristics of personality characteristics on household charitable donations under different levels of per capita family net income. Through the above series of empirical tests, the impact of personality characteristics on household charitable donation is analyzed, which provides a scientific basis for the analysis of household donation behavior.

The rest of this paper is structured as follows: part 2 review the prosocial behavior studies employing household charitable donation behavior and personality trait theory. Part 3 mainly introduces the definition, selection and statistical description of the research variables, and constructs the relevant models used in this empirical study. Part 4 starts from the analysis of one-way OLS regression, processing effect and other models, and uses the selected analytical personality to empirically test the effect of Chinese residents’ charitable donation behavior under this dimension of personality characteristics; And further carried out a series of heterogeneity analysis; The fifth part is conclusions and suggestions.

## Literature review

### Household charitable donation behavior

According to Kline et al’ [9] definition, prosocial behavior is the extent that it goes against one’s selfish interests and as a result potentially increases the payoffs to another person. The concept of prosociality is of fundamental importance to political Science, as it underlies motivations for political participation, social cooperation, charitable giving, voluntarism, and redistributive preferences. Charitable donation refers to the voluntary donation of resources to support those activities aimed at promoting social improvement without directly expecting economic returns [10]. Depending on the originator, charitable donations can be divided into individual donations, household donations, corporate donations, and national donations. Previous research on charitable donations has focused on cognitive and affective of individuals domains such as moral identity [11, 12], norms [13], guilt [14], sympathy [15], and happiness [16]. The household charitable donation behavior is a representative family prosocial behavior. It is now well understood that household donations are a major component of the total income of many charities, as for equivalent organizations overseas [17]. They are also an important way in which families express their support for the work of charitable organizations.

Household charitable donation is an activity of giving property voluntarily and gratuitously to the family for charitable purposes. Through micro-data tracking research, scholars’ empirical research on charitable donations found that the amount of donations is affected by family income [18], and according to the life course theory, with the growth of age, the more personal wealth accumulated, the higher the enthusiasm of donations may be [19]. Similarly, there is a positive relationship between education level and donation level [20]. The study found that family composition played a certain role, and the existing research evidence generally showed that married families, families with children, female-headed families and party member families made greater absolute contributions [18]. However, these do not fully explain the difference of household charitable donation behavior.

Economics usually uses utility maximization to study the behavior of household charitable donation, and finds that charitable donation can affect the utility in two positive ways: producing a good feeling of exerting self-worth [21, 22] and obtaining a good social image [23]. Both of these positive effects come from individuals’ implicit psychological factors, especially for homeowners who influence household donation behavior. In addition to family background and characteristics, there are few studies that discuss the impact of the personality of household head on household donation behavior.

### Big Five personality traits

The Big Five model promoted by Fiske [24] is a widely accepted personality trait model [25]. The term personality traits refers to stable sets of psycho-logical characteristics that uniquely influence people’s patterns in cognition, feelings and decision-making [26]. There are five labeled personality traits in the Big Five model: (1) Conscientiousness, showing the relationship between individual’s sense of achievement and the degree of dedication; (2) Agreeableness, highlighting the difficulty of cooperation between individuals and others; (3) Neuroticism refers to the tendency to experience negative emotions, such as anger, anxiety, and depression; (4) Openness, to a certain extent, reflects the individual’s innovation ability; (5) Extraversion represents an individual’s energetic engagement, sociability, as well as assertiveness.

Some studies have turned to the people-centred approach to examine the personality profile composition of people and discuss how these personality profiles affect people’s behavioural/decision-making patterns [25, 27, 28]. For example, household pro-environmental behaviors and prosocial behaviors. Wang et al. [29], for example, found that open-ness, agreeableness, and conscientiousness positively influence household energy-saving while neuroticism showed a negative impact. There is some evidence that prosocial tendencies are related to a person’s underlying personality traits. Kline et al. [9] investigate the effect of personality on prosocial political behavior in a Bayesian multilevel meta-analysis (MLMA) of 15 published, interdisciplinary experimental studies. The result find that the Big Five traits of Agreeableness and Openness are significantly and positively associated with prosocial behavior, while none of the other three traits are. Kanacri et al. [30] identify heterogenic longitudinal patterns of change in prosocial behavior from adolescence to early adulthood and their association with change in Big Five personality traits from adolescence until early adulthood. Foreign scholars have recognized the potential impact of personality characteristics on charity behavior: conscientiousness and neuroticism are inversely proportional to donation behavior, while openness has a positive impact on donor behavior. These findings show that the unobserved heterogeneity of individuals is very important in determining charitable behavior. However, there is currently a lack of evidence from China.

## Materials and methods

### Data and sample

The data used in the empirical part of this paper is from the "China Family Panel Studies" project (CFPS) implemented by the China Social Sciences Research Center of Peking University in 2018. CFPS is a multi-level, multi-dimensional, and long-term comprehensive tracking survey project that involves data at the individual, family, and community levels, reflecting changes in China’s society, economy, population, education, and health [31]. The CFPS, a national representative survey, is a representative social survey conducted nationwide biennially since 2010 to better understand social change [32]. The 2018 survey covers 14960 households in 25 provinces/cities/autonomous regions in mainland China except Tibet, Qinghai, Ningxia, Xinjiang and Inner Mongolia.

### Measures

#### Dependent variable

Through sorting out the existing questionnaire information in the Chinese family tracking survey, household charitable donations include three kinds of donors: relatives, others and society. To analyze the influence of the personality characteristics of the household heads on the household charitable donation behavior in detail, this paper uses the household charitable donation behavior and the household charitable donation level to carry out the research. This article refers to Wang and Li [32] who measured with two indicators, including whether household charitable donation occurs and the specific amount families gave in the past 12 months. The incidence of making a donation is a binary variable, which is coded as one if the household head donated in the past 12 months and 0 otherwise. The amount of charitable giving is a continuous variable, and the log transformation of the amount of donation is used in the multivariate analysis to reduce the skewness of the distribution.

#### Independent variables

This paper uses the "Big Five" personality classification method commonly used by the academic community to measure the personality characteristics variables of the household heads. In the 2018 CFPS, a questionnaire specifically aimed at the personality characteristics of adult respondents was added. Based on the theoretical basis of NEO-PI-R, a widely used "Big Five" personality measurement tool, and the Chinese adjective “Big Five” Personality Scale (BFFP-CAS), the questionnaire constructed a five-dimensional investor personality characteristics scale based on the CFPS questionnaire, including openness, conscientiousness, extraversion, agreeableness and neuroticism. This paper takes the average score of each dimension of “Big Five” Personality Scale (BFFP-CAS), to measure the score of the five dimensions of the personality characteristics of the household heads.

#### Covariates

According to the research of domestic and foreign scholars on the influencing factors of residents’ household charitable donation behavior, this paper mainly controls the family characteristic variables and the individual characteristics of the household heads when analyzing the household charitable donation behavior. The control variables of family characteristics include family size, proportion of children, per capita family net income, family net assets, and whether the family owns house. The control variables of household head’s individual characteristics include the head’s gender, age, registered residence, marital status, work situation, years of education, religious belief, intellectual level and health level.

#### Statistical analysis

The data used in this paper is the household, adult and child questionnaire data in the CFPS database in 2018, and the sample covers 25 provinces/cities/autonomous regions. A total of 10479 samples were obtained after eliminating the samples with missing values in the main variables. As shown in Table 1, in the household charitable donation behavior, the proportion of families with external donation behavior reached 40.3% of the total sample, and the logarithmic mean value of donation amount was 2.854, among which the proportion of families Donation to relatives was 25.6%, and the logarithmic mean value of donation amount was 2.001; Secondly, the proportion of families Donation to society is 21.7%, and the logarithmic mean value of donation amount is 0.679; The proportion of families Donation to others is relatively small, 9.9%, and the logarithmic mean value of donation amount is 1.163.

**Table 1:** Variable definition and descriptive statistics.

Variables		Questionnaire questions	Mean	SD
Dependent variable	External donation behavior	External donations greater than zero = 1, others = 0	0.403	0.491
	External donation level	Add up the amount of external donations, and then take the logarithm	2.854	3.659
	Donation to relatives	Donation to relatives greater than zero = 1, others = 0	0.256	0.437
	Donation level to relatives	Add up the amount of donations to relatives, and then take the logarithm	2.001	3.472
	Donation to others	Donation to others greater than zero = 1, others = 0	0.099	0.299
	Donation level to others	Add up the amount of donations to others, and then take the logarithm	0.679	2.086
	Donation to society	Donation to society greater than zero = 1, others = 0	0.217	0.412
	Donation level to society	Add up the amount of donations to society, and then take the logarithm	1.163	2.291
Independent variable	Conscientiousness	Rigorous and serious	3.904	0.636
		Often very lazy		
		Be efficient		
	Extraversion	Talkative	3.377	0.704
		Be outgoing and sociable		
		Implicit and conservative		
	Agreeableness	Sometimes rude to others	3.845	0.602
		The nature is relatively tolerant		
		Considerate of others		
	Openness	with Originality	3.138	0.869
		Attach importance to artistic experience and aesthetics		
		Be imaginative		
	Neuroticism	Often worried	2.955	0.749
		Easy to be nervous		
		Can handle pressure well		
Control variable	Family size	Total household population	3.670	1.921
	Proportion of children	Number of children in the family/total family population	0.128	0.176
	Logarithm of per capita household net income	Logarithm of per capita household net income	9.638	1.102
	Logarithm of household net assets	Logarithm of household net assets	11.994	3.894
	Whether the family has its own house	Own housing = 1, others = 0	0.856	0.352
	Gender of household heads	Male = 1, female = 0	0.537	0.499
	Age of household heads	Age of household heads	51.709	14.097
	Census register of household heads	Non-agricultural census register = 1, others = 0	0.476	0.499
	Marital status of household heads	Married or cohabiting = 1, others = 0	0.843	0.364
	Work status of household heads	In employment = 1, others = 0	0.757	0.429
	Education years of household heads	Accumulated years of education of household heads	7.276	4.829
	The religious belief of household heads	Is it a member of a religious belief group (yes = 1, not = 0)	0.036	0.185
	Intelligence level of household heads	household heads’ intelligence level test score	4.987	1.425
	Health level of household heads	Physical condition (from average to better, score 1 to 5)	3.170	1.224

It shows that household external donations tend to be among familiar relatives, and direct donations to society are relatively cautious and small. Among the personality characteristics of the household heads, conscientiousness is the strongest, followed by agreeableness, extraversion, openness and neuroticism. Relatively speaking, the household heads with high extraversion lack openness, while the high agreeableness is more prominent in the low extraversion. The descriptive statistical results are basically consistent with the findings of the existing literature on the personality characteristics of the household heads. Among the individual characteristics and family characteristics of the sample household heads, the average family size is 3.67, of which the average proportion of children is 12.8%. The logarithm of the per capita net income of the family is 9.63, which is lower than the logarithm of the net assets of the family. Most families have their own house. 53% of the household heads surveyed are male, the average age is 51.7 years old, 47% are non-agricultural registered residence, 84.3% are married, and 75.7% are in employment, The average education years of household heads is 7.27, 3.6% of the household heads have religious beliefs, the average score of household heads’ intelligence level is 4.98 points, and the average score of the household heads’ health is 3.17 points.

### Model selection

#### Regression model

To examine the effect of personality characteristics on household charitable donation behavior, this paper selects household charitable donation behavior as dependent variable. First, the Probit model is used to empirically test whether the personality characteristics affect the various household donation behaviors.


$$y_{i}^{*}=\alpha +\beta x_{i}+\gamma h_{i}+\varepsilon_{i},\;y_{i}=\left\{ \begin{array}{c} 1,\;y_{i}^{*}>0\\ 0,\;y_{i}^{*}\leq 0 \end{array}\right.$$ (1)

In Formula (1), $y_{i}^{*}$ is an unobserved potential variable, ***y***_***i***_ is observed value of whether there is household donation behavior, ***x***_***i***_ is personality characteristic variable, including openness, conscientiousness, extraversion, agreeableness and neuroticism. ***h***_***i***_ is a series of control variables, including variables of household head individual characteristic and family characteristic.

Second, Tobit model is used to empirically test the influence of personality characteristics on the level of household external donations.


$$y_{i}^{*}=\alpha +\beta x_{i}+\gamma h_{i}+\varepsilon_{i},\;y_{i}=\left\{ \begin{array}{c} y_{i}^{*},\;y_{i}^{*}>0\\ 0,\;y_{i}^{*}\leq 0 \end{array}\right.$$ (2)

In Formula (2), $y_{i}^{*}$ is an unobserved potential variable, ***y***_***i***_ is observed value of the logarithm of the amount of household donations, ***x***_***i***_ is personality characteristic variable, including openness, conscientiousness, extraversion, agreeableness and neuroticism. ***h***_***i***_ is a series of control variables, including individual characteristic variables of household head and family characteristic variables.

#### Treatment effect model

First, take the mean value of personality characteristics of all households in the valid sample, and divide the family sample into two groups according to the mean value: (1) high openness personality group (openness_high = 1); (2) Low openness personality group (openness_low = 0).

In view of the existence of interference items in the actual sample rules, the "high openness personality characteristics" group cannot be effectively identified, so in this study, a latent variable is assumed and the selection equation is used to capture and distinguish.


$$Y=\beta^{\prime }X+\theta D+\mu$$ (3)

In Formula (3), ***X*** contains the intercept term and a set of exogenous explanatory variables that affect ***Y***. The indicative variable ***D*** can be regressed by the following binary choice model:

$$D^{*}=\alpha_{0}^{‘}Z+\alpha_{1}^{’}X+v$$ (4)

In Formula (4), D* represents the latent variable mentioned above. If D*≥0, then ***D***, otherwise D*<0, then ***D***. Z is an exclusive constraint variable that needs to be included in the choice model without entering the regression equation.

#### PSM model

This paper use propensity score matching method to test the robustness of the benchmark regression results. Specifically, by defining the two-dimensional dummy variable ***D*** = {**0**, **1**}, the average value of openness personality characteristics of household heads was divided into treatment group and control group. ***D*** = **1**, treatment group, the household head is highly openness, ***D*** = **0**, control group, the household head is low openness. ***y***_**1*i***_ and ***y***_**0*i***_ is the financial assets selection of households in the treatment group and the control group respectively. The average treatment effect of the treatment group is:

$$ATT=E[y_{1i}|D_{i}=1]-E[y_{0i}|D_{i}=0]$$ (5)

In Formula (5), ***E***[***y***_**1*i***_|***D***_***i***_ = **1**] is the household charitable donation behavior of the household head with high openness personality characteristics; ***E***[***y***_**0*i***_|***D***_***i***_ = **0**] is the household charitable donation behavior of the household head with low openness personality characteristics.

#### Threshold regression

Under different per capita household net income, personality characteristics may also have a nonlinear effect on the level of household charitable donations. Therefore, the following panel threshold model:

$$Y_{i}=\gamma_{0}+\gamma_{1}X_{i}\times I\;({Adj}_{i}\leq \theta )+\gamma_{1}X_{i}\times I\;({Adj}_{i}>\theta )+\gamma_{c}Z_{i}+\mu_{i}+\varepsilon_{i,\;t}$$ (6)

In Formula (6), ***Adj***_***i***_ is threshold variable. ***I***(∙) is an indicator function with value is 0 or 1 (If the condition in brackets is met, it is 1, otherwise it is 0). Formula (3) is a single threshold model, which can be extended to multi-threshold cases according to the empirical results.

## Results

### Estimation results of regression model

This paper first uses Probit model and Tobit model to conduct a large sample quantitative analysis of the impact of openness, conscientiousness, extraversion, agreeableness and neuroticism on household donation behavior. Table 2 shows the main estimated results of the benchmark regression. The model (1) (2) uses Probit model to test the influence of the household heads personality characteristics on whether the family has donation behavior, and the model (3) (4) uses Tobit model to test the influence of the household heads personality characteristics on the various household donation levels. The results of regression estimation show that the influence coefficients of openness and conscientiousness of the household heads on household charitable donation behavior and donation level are statistically positive at 0.01 significance level. This shows that for Chinese household donation behavior, openness personality has a positive effect on household donation behavior.

**Table 2:** Estimated results of the impact of personality characteristics on household external donations.

Variables	(1)	(2)	(3)	(4)
	External donation behavior	External donation behavior	External donation level	External donation level
Conscientiousness	0.0406*	0.0820***	0.268*	0.523***
	(1.92)	(3.64)	(1.78)	(3.66)
Extraversion	-0.0178	0.0255	-0.155	0.151
	(-0.96)	(1.32)	(-1.20)	(1.24)
Agreeableness	0.0487**	0.0307	0.373**	0.216
	(2.23)	(1.35)	(2.43)	(1.49)
Openness	0.111***	0.0765***	0.874***	0.549***
	(7.49)	(4.91)	(8.27)	(5.46)
Neuroticism	-0.0398**	0.00375	-0.315***	0.0544
	(-2.35)	(0.21)	(-2.66)	(0.47)
The religious belief of household heads		0.0975***		0.650***
		(3.39)		(3.56)
Family size		-0.00698		-0.0568
		(-0.84)		(-1.09)
Proportion of children		0.284***		1.770***
		(3.23)		(3.27)
Net household income per capita		0.233***		1.721***
		(12.88)		(17.50)
household net assets		0.00936**		0.0675***
		(2.50)		(2.96)
Marital status of household heads		0.158***		1.254***
		(4.05)		(5.08)
Work status of household heads		0.0969***		0.528**
		(2.87)		(2.47)
Gender of household heads		-0.0937***		-0.503***
		(-3.41)		(-2.89)
Age of household heads		-0.00708***		-0.0435***
		(-6.00)		(-5.82)
Education years of household heads		0.0252***		0.167***
		(7.50)		(7.92)
The religious belief of household heads		0.0880		0.651
		(1.25)		(1.50)
Intelligence level of household heads		0.0391***		0.267***
		(4.04)		(4.28)
Whether the family has its own house		-0.0716*		-0.471**
		(-1.86)		(-1.96)
Health level of household heads		0.0226**		0.110
		(2.00)		(1.52)
_cons	-0.762***	-3.646***	-4.896***	-25.54***
	(-5.96)	(-14.97)	(-5.39)	(-17.84)
var (e. external donation level)			60.49***	51.05***
			(39.02)	(39.32)
N	10479	10479	10479	10479

*p < 0.01

**p < 0.005

***p < 0.001

### Robustness check

#### Estimated results of treatment effect model

According to the empirical results of benchmark regression, among the many factors influencing the current household charitable donation behavior in China, divided by the non-cognitive ability in the "Big Five" personality characteristics, which has an important impact on the household donation behavior, the innovative personality characteristics represented by openness also have an important impact on the current household donation behavior in China. However, from the sample size and robustness test results, the simple OLS identification strategy constructed by benchmark regression has many disadvantages: it is unable to effectively deal with the interference effect of "selective bias" in the stage of personality characteristics impact analysis, which ultimately led to the inability to conduct causal effect analysis on the impact of personality characteristics in household charitable donation behavior in a real sense. Based on this problem, this section further uses the maximum likelihood estimation method to measure and analyze the causal effect of personality characteristics on household charitable donation behavior, taking openness personality characteristics as the starting point.

Based on the above analysis, and considering that the residents’ risk preference consciousness and openness personality characteristics are both innovative personality attributes [33], the average value of risk preference (openness_ave) of the household heads in the same community/village where a family is located (excluding itself) is used as the proxy variable in actual operation. First, the average value of the risk preference of the household heads in the same community/village where a family is located (excluding itself) meets the strict exogeneity. Second, Since the household charitable donation behavior is affected by the same group effect, the household heads with similar personality characteristics often have more consistency in the performance of relevant donation behavior, which to some extent meets the relevant requirements. Therefore, the exclusive constraint variable selected in this paper will not have a direct impact on the dependent variable, but can only have an indirect impact through the latent variable, so the exclusive constraint is satisfied.

As shown in Table 3, after accurately selecting the control variables such as individual characteristics and family characteristics of other household heads, using risk preference as the choice variable of openness personality characteristics, we can clearly analyze the positive effect statistical results at the 0.05 significance level, and this conclusion can be effectively supported by the regression equation or selection equation to estimate the correlation coefficient of error items. It can be further concluded that residents with openness personality tend to be more willing to make charitable donations, that is, there is a strong positive causal relationship between openness personality and charitable donations. The reasons for this may include: (1) Open residents tend to express their true thoughts more efficiently, and to some extent, they are regarded as the typical temperament of adventurers and doers; (2) Based on the friendliness of personality, open residents can often establish a closer friendly and mutual relationship with the surrounding people. Therefore, through a wide range of social groups, they can help others get better light and heat effects, and thus establish a good social image.

**Table 3:** Estimated results of the impact of openness personality characteristics on household external donations (treatment effect).

Variables	(1)	(2)	(3)	(4)
	External donation behavior	External donation behavior	External donation level	External donation level
Main	Table(A): Regression equation			
High Openness	0.479***	0.330**	3.194***	2.929***
	(4.89)	(2.31)	(4.79)	(3.04)
Conscientiousness	0.0188**	0.0303***	0.130**	0.209***
	(2.32)	(3.87)	(2.15)	(3.63)
Extraversion	-0.00483	0.00970	-0.0540	0.0580
	(-0.68)	(1.43)	(-1.03)	(1.16)
Agreeableness	0.0193**	0.0110	0.166***	0.0874
	(2.30)	(1.37)	(2.66)	(1.47)
Neuroticism	-0.0141**	0.00134	-0.136***	0.0235
	(-2.17)	(0.21)	(-2.80)	(0.50)
Control variable	NO	YES	NO	YES
_cons	0.0696	-0.793***	0.663	-7.444***
	(1.00)	(-7.58)	(1.33)	(-10.04)
High Openness	Table(B): Choice equation			
Mean of risk preference	0.105***	0.0912***	0.107***	0.0893***
	(5.10)	(4.11)	(5.14)	(4.07)
_cons	-0.150***	-0.127***	-0.153***	-0.123***
	(-4.12)	(-3.26)	(-4.14)	(-3.18)
athrho	-0.534***	-0.391**	-0.452***	-0.471***
	(-4.18)	(-2.01)	(-3.90)	(-2.63)
lnsigma	-0.634***	-0.719***	1.353***	1.296***
	(-17.34)	(-16.29)	(45.24)	(27.75)
N	10479	10479	10479	10479

*p < 0.01

**p < 0.005

***p < 0.001

#### Estimated results of propensity score matching method

To ensure the reliability of the core conclusions and better reveal the causal relationship between personality characteristics and household charitable donations, this paper also uses the propensity score matching (PSM) method for robustness testing. First, estimate the probability model of the high-low openness personality characteristics of household heads, and get the tendency score in the sample. Then, the nearest neighbor matching method in the caliper is used to select household samples with the same or similar household characteristics from the control group for the treatment group. In addition to the different values of the variables of the openness personality characteristics, the other characteristic variables of the matched families are the same. Based on the consistency of the two sets of covariates after being matched, Average treatment effect (ATT) of the two variables of external donation behavior and external donation level is calculated. The grouping strategy and average effect estimation method based on the average score of open personality characteristics can effectively reduce the estimation bias caused by self-selection.

**Fig 1:**
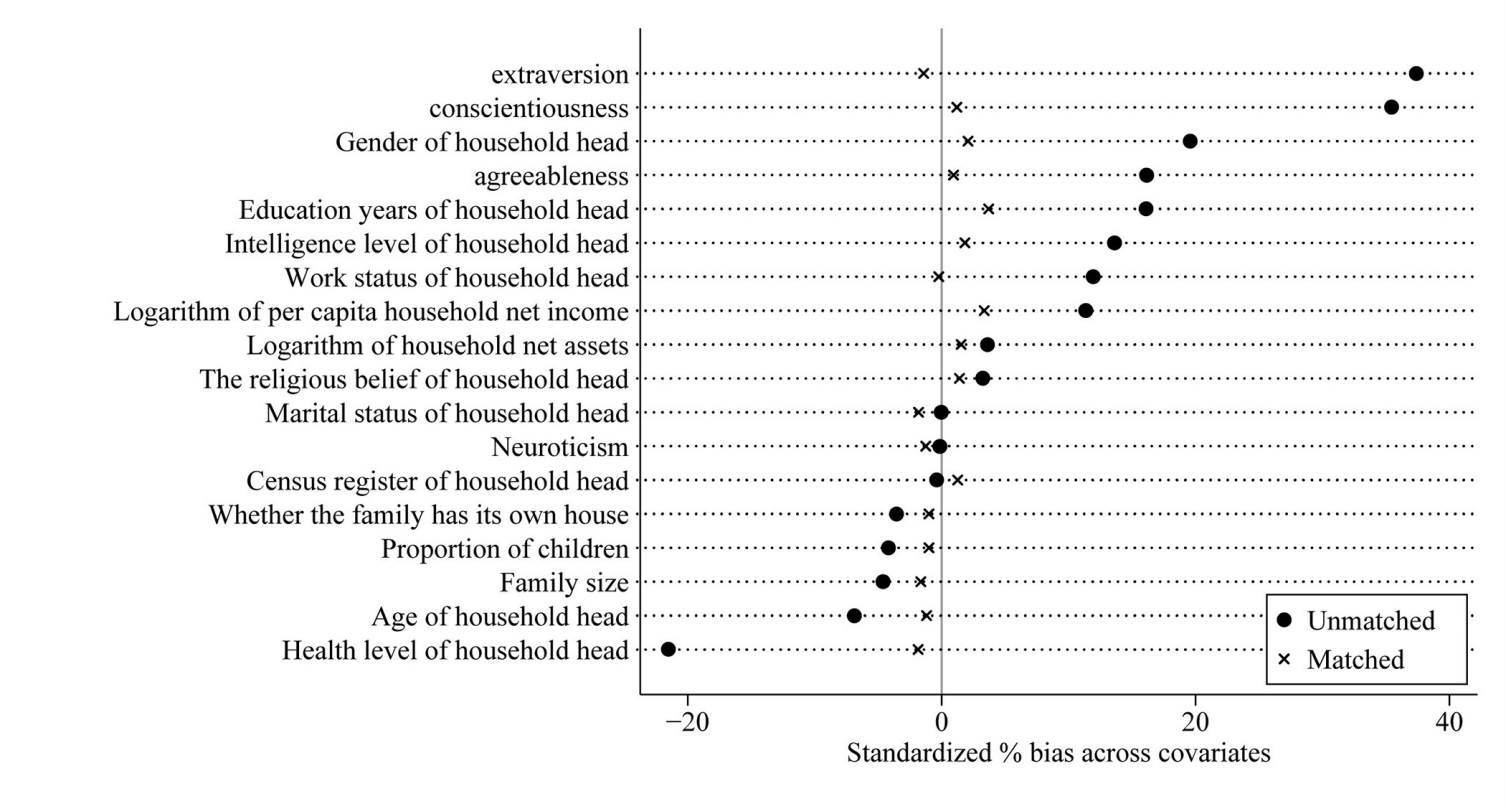
Standardized deviation of each variable before and after being matched.

To ensure the quality of matching results, it is necessary to ensure the balance of variables in the processing group and control group. After matching, there should be no significant differences in the control variables in the family characteristics and the individual characteristics of the household heads, except for the differences in the family financial asset selection and other related dependent variables between the high openness personality group and the low openness personality group. Therefore, the balance test was carried out for each control variable in the family of the high openness personality group and the low openness personality group. The test results are shown in Fig 1, and the standardized deviation of each variable decreased after being matched.

As shown in Table 4, by calculating the average treatment effect (ATT) of the two variables of external donation behavior and external donation level, it is found that there are certain differences between the two dependent variables before and after being matched. The PSM method controls the endogenous problems caused by the selection of independent variables. The PSM method controls the endogenous problems caused by the selection of independent variables. According to the robust estimation results in Table 4, without considering the "selective bias" of individual samples, the influence effects of all variables are statistically positive at the 0.01 significance level. Therefore, the innovative personality characteristics represented by openness have a significant positive causal relationship with household donation behavior.

**Table 4:** Causality results of high-low openness personality characteristics on household charitable donation behavior.

Variables	Matching type	High Openness	Low openness	Difference value	S.E.	T-stat
		Personality characteristics group	Personality characteristics group			
External donation behavior	Before being matched	0.438	0.368	0.070***	0.010	7.35
	After being matched	0.438	0.391	0.046***	0.010	4.41
External donation level	Before being matched	3.155	2.540	0.615***	0.071	8.63
	After being matched	3.153	2.727	0.427***	0.077	5.51

*p < 0.01

**p < 0.005

***p < 0.001

### Further research: Heterogeneity analysis

#### Differences of household charitable donations level

This section intends to use the quantile regression method to supplement the empirical test of the research on the impact of personality characteristics on household charitable donations. Specifically, the five representative quantiles of 10%, 25%, 50%, 75% and 90% are selected for estimation. In this part, the sample with non-zero amount of household charitable donations is selected for estimation. In addition, as shown in Table 5, this section also uses mixed OLS regression to verify the effectiveness of OLS regression analysis results.

**Table 5:** Estimated results of the impact of personality characteristics on the household charitable donations level (quantile regression).

Variables	(1)	(2)	(3)	(4)	(5)	(6)
	OLS	QR_10	QR_25	QR_50	QR_75	QR_90
Conscientiousness	-0.015	-0.075	-0.066	-0.059	0.002	0.108*
	(0.050)	(0.098)	(0.084)	(0.060)	(0.054)	(0.059)
Extraversion	-0.029	-0.081	-0.089	-0.029	-0.002	0.007
	(0.040)	(0.080)	(0.069)	(0.049)	(0.044)	(0.049)
Agreeableness	0.040	0.023	-0.013	0.063	0.058	0.036
	(0.048)	(0.098)	(0.084)	(0.060)	(0.054)	(0.060)
Openness	0.145***	0.271***	0.262***	0.092**	0.097**	0.036
	(0.034)	(0.069)	(0.060)	(0.043)	(0.038)	(0.042)
Neuroticism	0.050	0.095	0.134**	0.014	0.029	0.023
	(0.038)	(0.077)	(0.066)	(0.047)	(0.042)	(0.047)
_cons	1.470***	-0.739	0.188	1.146*	2.534***	4.170***
	(0.562)	(0.965)	(0.829)	(0.592)	(0.531)	(0.587)
N	4227	4227	4227	4227	4227	4227

The values in brackets are t-value

*: p < 0.01

**: p < 0.005

***: p < 0.001; Column (1) is the OLS regression results of personality characteristics on the level of household charitable donation, and columns (2) to (6) are the regression results of household charitable donation at five quantiles.

From the results, among the families with charitable donation behavior, the openness personality characteristics have a robust and significant positive impact on household charitable donation behavior, indicating that the results of the benchmark regression model are robust. With the improvement of the level of household charitable donation, the positive effect of the head of household’s openness personality on the household charitable donation behavior has gradually weakened, and becomes insignificant at the 90% quantile. To some extent, it shows that for ordinary residents with low level of charitable donation, the household heads with openness personality is easier to understand charitable donation. However, with the continuous improvement of donation level, it is necessary to cooperate with conscientious personality characteristics to assist in upgrading.

#### Differences of household donations type

To analyze the impact of the personality characteristics of the household heads on the household charitable donation behavior in detail, this part is divided into three types of donation behavior to conduct research. First, Probit model and Tobit model are used to conduct a large sample quantitative analysis of the effects of openness, conscientiousness, extraversion, agreeableness, and neuroticism on household donation behavior. Table 6 shows the main estimation results of the benchmark regression. Among them, the model (1) (3) (5) uses Probit model to test the influence of the personality characteristics of the household heads on the household donation behavior, and the model (2) (4) (6) uses Tobit model to test the influence of the personality characteristics of the household heads on the household donation amount.

**Table 6:** The estimated results of the effect of personality characteristics on household classified charitable donation.

Variables	(1)	(2)	(3)	(4)	(5)	(6)
	Donation to relatives	Donation level to relatives	Donation to others	Donation level to others	Donation to society	Donation level to society
Main						
Conscientiousness	0.0490**	0.473**	0.0500	0.563	0.0817***	0.528***
	(2.03)	(2.15)	(1.61)	(1.60)	(3.18)	(3.12)
Extraversion	0.00937	0.101	0.0139	0.137	0.0150	0.107
	(0.45)	(0.55)	(0.53)	(0.46)	(0.70)	(0.75)
Agreeableness	0.0370	0.370*	-0.00115	0.0128	0.0498*	0.340**
	(1.50)	(1.66)	(-0.04)	(0.04)	(1.93)	(1.98)
Openness	0.0716***	0.645***	0.124***	1.454***	0.0568***	0.431***
	(4.28)	(4.17)	(5.87)	(5.76)	(3.23)	(3.61)
Neuroticism	0.0321*	0.283	0.0293	0.327	0.00708	0.0550
	(1.65)	(1.61)	(1.21)	(1.16)	(0.35)	(0.41)
Control variables	YES	YES	YES	YES	YES	YES
_cons	-3.738***	-36.09***	-4.117***	-48.18***	-4.271***	-30.44***
	(-14.22)	(-16.05)	(-12.84)	(-12.94)	(-15.76)	(-17.33)
var (e. Donation level to relatives)		96.42***				
		(30.14)				
var (e. Donation level to others)				137.8***		
				(18.01)		
var (e. Donation level to society)						50.72***
						(27.63)
N	10479	10479	10479	10479	10479	10479

The values in brackets are t-value

*: p < 0.01

**: p < 0.005

***: p < 0.001

The influence coefficient of openness personality characteristics on the behavior and level of families under different types of donations is statistically positive at the 0.01 significance level, which indicates that openness personality characteristics have obvious positive effect on household donations. This shows that the innovative personality characteristic represented by openness is more important, and its marginal contribution to household donation behavior is more significant. In addition, conscientious personality has a significant positive impact on household donation to relatives and household donation to society. The possible reason is that conscientious individuals have high initiative and self-discipline, and correspondingly have a sense of responsibility to help others.

#### Differences of net household income per capita

The impact of personality characteristics on the level of household charitable donation may be different under different levels of per capita family net income. To reveal the possible nonlinear impact of personality characteristics on the level of household charitable donation, this paper takes per capita family net income as the threshold variable to conduct threshold effect regression analysis, and the results of the regression test are shown in Table 7.

**Table 7:** Test results of threshold effect.

Order	Threshold	SSR
3	9446	1.211e+05
1	24570	1.217e+05
2	49990	1.213e+05
4	52187	1.209e+05

Table 7 shows that the F statistic of the four threshold characteristics of household charitable donation level has passed at 0.01 significance test level, while the first three threshold values differ greatly. Therefore, this paper uses the triple threshold model to test the threshold effect of personality characteristics on the level of household charitable donation under different per capita household net income levels. The results are shown in Table 8. With per capita family net income increasing, the impact of openness personality on the level of household charitable donation becomes significant and the coefficient increases, which indicates that the impact of openness personality of the household heads on the level of household charitable donation has a nonlinear characteristic of "marginal effect" increasing. At the same time, when the family net income reached a certain level, the conscientious personality characteristics showed a similar effect.

**Table 8:** Estimated results of the effect of personality characteristics on household charitable donation behavior (threshold effect).

Variables	(1)	(2)	(3)	(4)
Range of households per capita net income	(0,9446]	(9446,24570]	(24570,49990]	(49990,∞)
Conscientiousness	0.068	0.147	0.265**	0.618***
	(0.099)	(0.094)	(0.129)	(0.175)
Extraversion	0.053	0.003	0.031	0.160
	(0.092)	(0.082)	(0.108)	(0.140)
Agreeableness	0.102	0.096	0.097	-0.054
	(0.104)	(0.097)	(0.133)	(0.169)
Openness	0.118*	0.262***	0.239***	0.401***
	(0.070)	(0.066)	(0.092)	(0.123)
Neuroticism	0.152*	-0.065	0.026	0.053
	(0.086)	(0.076)	(0.100)	(0.128)
Control variable	YES			
_cons	-2.569***	-2.044**	-2.054**	-3.034**
	(0.829)	(0.854)	(1.030)	(1.244)

*p < 0.01

**p < 0.005

***p < 0.001

#### Differences of age of household heads

According to the 25%, 50% and 75% of the age variable of the household heads in the sample, this paper sets the age range of the household heads: age≤42, 42<age≤52, 52<age≤62, and 62<age, in order to examine the impact of the characteristics of household ownership on the household donation behavior under different age ranges, as shown in the Table 9. The influence of the personality characteristics of the household heads on household donation behavior has obvious life cycle characteristics. The influence of the personality characteristics of the household heads on household donation behavior has obvious life cycle characteristics. However, for households over 52 years old, conscientious personality has a significant impact on household donation behavior. It shows that with the growth of age, donation behavior begins to become a responsibility.

**Table 9:** The estimated results of the impact of personality characteristics on household charitable donation behavior under the four-age group.

Variables	(1)	(2)	(3)	(4)
Age range of household heads	age≤42	42<age≤52	52<age≤62	62<age
Conscientiousness	0.094	0.150	0.287**	0.224**
	(0.74)	(1.31)	(2.47)	(2.26)
Extraversion	-0.00178	-0.0260	0.132	0.120
	(-0.02)	(-0.26)	(1.30)	(1.34)
Agreeableness	0.0248	0.0821	0.105	0.103
	(0.19)	(0.68)	(0.89)	(0.98)
Openness	0.301***	0.233***	0.286***	0.228***
	(3.10)	(2.91)	(3.84)	(3.30)
Neuroticism	0.070	-0.071	-0.037	0.091
	(0.69)	(-0.71)	(-0.39)	(1.11)
_cons	-8.254***	-6.854***	-7.158***	-7.333***
	(-5.64)	(-4.15)	(-3.95)	(-5.86)
N	2476	2657	2554	2792

*p < 0.01

**p < 0.005

***p < 0.001

## Discussion

In the context of the country’s efforts to promote the strategy of common prosperity, as a supplementary form of redistribution, it is beneficial to actively carry out the three distributions through household charitable donations and other activities to help the poor and the weak. From the perspective of the main sponsors of charity, in addition to individuals, enterprises, and the country, there is also an important component—the family as the unit. The charitable donation behavior of families reflects their pro-social tendencies. Existing research on the relationship between personality traits and prosocial behavior has proposed that it is also possible that the personality characteristics will change with the change of cognition caused by past experience in different time and environment [9, 34]. This study uses the data of the CFPS project to conduct empirical analysis to test the impact of the "Big Five" personality characteristics of household heads on household donation behavior. The results show that, after controlling the individual characteristics and family characteristics of the household heads, the openness and conscientiousness of the household head personality have a similar positive effect on the household charitable donation behavior and donation level with that of developed countries. Similar to family characteristics and individual characteristics of the household head, non-objective factors such as personality characteristics of residents have the same economic effect on household charitable donation. On this basis, this paper focuses on openness personality characteristics, and tests the causal effect of openness personality characteristics on household donation behavior through processing effect model and propensity score matching method. This article complements the theoretical study of the Big Five personality on household charitable donation behavior.

Based on these findings, three targeted behavioral intervention strategies are formulated to promote household charitable donation behaviors. First, at the level of the national government, personality education can be incorporated into the existing education system [35], especially in the process of teenagers’ personality formation. Continue to promote the improvement of residents’ income level, and actively play the role of the openness personality characteristics of the heads of wealthy households in increasing the effect of charitable donations. Secondly, charitable organizations should be open, transparent and efficient in charitable activities, so as to stimulate households with conscientious personality characteristics to improve the social charitable donations level. Charity organizations should also strengthen Internet charity promotion and donation facilitation measures, so that the head of household with openness personality characteristics can easily participate in charity donation activities, and promote the implementation of three distributions. Finally, individual household heads should actively shape stable and openness personality characteristics. The head of household should maintain his enthusiasm for charity and public welfare as he grows older. At the same time, through the civilized construction of the community/village environment, cultivate a positive social style, promote the formation of openness personality characteristics of residents, and improve the donation efficiency of the next three distributions of common prosperity.

There are some limitations of this study, including the use of a self-report measure of human personality. Human personality traits are difficult to obtain in practice, and future research would benefit from the acquisition of big data and machine learning techniques. There are also incomplete controlled biases or other confounding factors that are not discussed in our research, such as household self-construal, net household expenditure. We also do not explore possible mechanisms involved in the potential effect of human personality change on household charitable donation behavior and did not introduce the comprehensive function of personalities. The survey in this study was conducted in mainland China. Personality profile characteristics can be different in different locations and cultures, and thus the applicability of the findings in other regions and countries require more investigation.

## Conclusion

This study selected CFPS family questionnaire data as a sample to explore the relationship between personality characteristics and household donation behavior. The results show that after controlling the individual characteristics and family characteristics of the head of household, the openness personality has a significant positive impact on the household external donation behavior, and the conscientious personality has a significant positive impact on the household donation to society. In the further study of difference of donation types, it is found that the innovative personality characteristics represented by openness are more important, and its marginal contribution to household donation behavior is more significant. In the rising change of per capita household net income, the impact of the openness personality of the household head on the level of household charitable donation has the nonlinear characteristic of "marginal effect" increasing. The influence of the personality characteristics of the household heads on the household donation behavior has obvious life cycle characteristics, and the openness personality characteristics have obvious positive impact on the household donation behavior of the household heads at different ages stage. For households over 52 years old, conscientious personality has a significant impact on household donation behavior. Based on analysis and discussion, the study suggests that national governments, charitable organizations, and individual household heads adopt targeted personality interventions to encourage household charitable donations. Researchers have called for the wider application of a human centered approach that links personality characteristics from different cultural backgrounds to family prosocial behavior research.
